# Evaluation of Cross-Immunity to the Mpox Virus Due to Historic Smallpox Vaccination

**DOI:** 10.3390/vaccines11101541

**Published:** 2023-09-28

**Authors:** Giulia Matusali, Elisa Petruccioli, Eleonora Cimini, Francesca Colavita, Aurora Bettini, Eleonora Tartaglia, Settimia Sbarra, Silvia Meschi, Daniele Lapa, Massimo Francalancia, Licia Bordi, Valentina Mazzotta, Sabrina Coen, Klizia Mizzoni, Alessia Beccacece, Emanuele Nicastri, Luca Pierelli, Andrea Antinori, Enrico Girardi, Francesco Vaia, Alessandro Sette, Alba Grifoni, Delia Goletti, Vincenzo Puro, Fabrizio Maggi

**Affiliations:** 1 Laboratory of Virology and Biosafety Laboratories, National Institute for Infectious Diseases “Lazzaro Spallanzani” IRCCS, Via Portuense 292, 00149 Rome, Italy; giulia.matusali@inmi.it (G.M.); francesca.colavita@inmi.it (F.C.); aurora.bettini@inmi.it (A.B.); silvia.meschi@inmi.it (S.M.); daniele.lapa@inmi.it (D.L.); massimo.francalancia@inmi.it (M.F.); licia.bordi@inmi.it (L.B.); sabrina.coen@inmi.it (S.C.); klizia.mizzoni@inmi.it (K.M.); fabrizio.maggi@inmi.it (F.M.); 2 Translational Research Unit, National Institute for Infectious Diseases “Lazzaro Spallanzani” IRCCS, Via Portuense 292, 00149 Rome, Italy; elisa.petruccioli@inmi.it (E.P.); settimia.sbarra@inmi.it (S.S.); delia.goletti@inmi.it (D.G.); 3 Laboratory of Cellular Immunology and Farmacology, National Institute for Infectious Diseases “Lazzaro Spallanzani” IRCCS, Via Portuense 292, 00149 Rome, Italy; eleonora.tartaglia@inmi.it; 4 HIV/AIDS Unit, National Institute for Infectious Diseases “Lazzaro Spallanzani” IRCCS, Via Portuense 292, 00149 Rome, Italy; valentina.mazzotta@inmi.it (V.M.); andrea.antinori@inmi.it (A.A.); 5 Highly Contagious Infectious Diseases Unit, National Institute for Infectious Diseases “Lazzaro Spallanzani” IRCCS, Via Portuense 292, 00149 Rome, Italy; beccacece.alessia@gmail.com (A.B.); emanuele.nicastri@inmi.it (E.N.); 6 Unità Operativa Complessa (UOC) Transfusion Medicine and Stem Cell, San Camillo Forlanini Hospital, 00152 Rome, Italy; lpierelli@scamilloforlanini.rm.it; 7 Scientific Direction, National Institute for Infectious Diseases “Lazzaro Spallanzani” IRCCS, 00149 Rome, Italy; enrico.girardi@inmi.it; 8 General Direction, National Institute for Infectious Diseases “Lazzaro Spallanzani” IRCCS, 00149 Rome, Italy; francesco.vaia@inmi.it; 9 Center for Infectious Disease and Vaccine Research, La Jolla Institute for Immunology, La Jolla, CA 92037, USA; alex@lji.org (A.S.); agrifoni@lji.org (A.G.); 10 Department of Medicine, Division of Infectious Diseases and Global Public Health, University of California, San Diego (UCSD), La Jolla, CA 92093, USA; 11 Risk Management Unit, National Institute for Infectious Diseases “Lazzaro Spallanzani” IRCCS, Via Portuense 292, 00149 Rome, Italy; vincenzo.puro@inmi.it

**Keywords:** Mpox virus, Orthopoxvirus, smallpox-vaccine, adaptive immunity, neutralizing antibodies, T cell response

## Abstract

When the Mpox virus (MPXV) began spreading globally in 2022, it became critical to evaluate whether residual immunity from smallpox vaccination provided cross-protection. To assess the cross-immune response to MPXV, we collected serum samples (*n* = 97) and PBMCs (*n* = 30) from healthy-donors, either born before 1974 and reporting smallpox vaccination during childhood or born after 1975 and not vaccinated with Vaccinia virus (VACV)-based vaccines. We evaluated the levels of anti-MPXV IgG and neutralizing antibodies (Nabs) and the presence of a T cell response against MPXV. We found anti-MPXV IgG and Nabs in 60 (89.6%) and 40 (70.1%) vaccinated individuals, respectively. We observed a T cell response to Orthopoxviruses and MPXV peptide pools in 30% of vaccinated individuals. We thus show that a high proportion of subjects who received the smallpox vaccine 40 to 60 years ago have humoral cross-immunity, while the T-cell-specific response against MPXV was observed in a smaller group (30%) of vaccinated individuals. This study, combined with information on immunity developed during natural infection or the administration of current vaccines, will contribute to a better understanding of humoral and cellular responses against MPXV.

## 1. Introduction

The worldwide smallpox vaccination campaign led to the eradication of the smallpox [1] declared on 8 May 1980. First-generation and currently available vaccines consist of a closely related Orthopoxvirus (OPXV), vaccinia virus (VACV), and lead to cross-protective immunity against smallpox and other OPXVs [2]. Several studies demonstrated that both antibody and T-cell-specific responses are crucial to controlling OPXV infection [3,4,5,6,7,8,9]. The central role played by neutralizing antibodies (Nabs) was supported by the clinical experience during the smallpox outbreaks, in which the pre-exposure administration of Nabs helped in preventing smallpox or reducing symptoms, and the early-post exposure inoculation protected against lethal smallpox [7]. Moreover, a prospective study carried out in the 1970s showed that smallpox contacted by a Nab titer of 1:32 or greater resulted in protection against the disease [8]. The importance of T cells in protection and recovery from smallpox and other OPXVs has been first presumed based on the evidence of serious or fatal adverse events related to VACV-based vaccines in children with congenital T cell immunodeficiency disorders and in adults with advanced HIV infections [8,9]. A mouse model of VACV infection suggested that CD4+ T-cell-dependent anti-VACV antibody production plays an important role in clearing the virus following acute infection, while in the absence of antibodies, CD8+ T cells can contribute to protection against the disease [3]. It is believed that the smallpox vaccine confers lifelong protection against severe disease [10], and studies carried out after smallpox eradication tried to estimate the waning effect by measuring the levels of immunological markers at different time-points post-immunization [11,12,13,14,15,16,17,18,19,20,21]. Nowadays, after smallpox eradication and the discontinuation of the vaccination campaign, the human population is largely unvaccinated. 

When the Mpox virus (MPXV), a zoonotic virus belonging to the Orthpoxvirus genus, started spreading worldwide, causing the 2022 multi-country outbreak, it became critical to determine whether residual immunity from historic smallpox vaccination conferred Mpox cross-protection and whether a vaccination campaign targeting at-risk individuals was required to halt MPXV transmission. In the macaque model [22], a vaccinia-specific B-cell response was demonstrated to be essential and sufficient for the protection of macaques from MPXV. In the same model, the passive transfer of human vaccinia-neutralizing antibodies protected non-immunized macaques from severe Mpox disease. According to studies conducted in the 1980s in the Democratic Republic of the Congo, the level of cross-protection against MPXV conferred by previous vaccination against smallpox was estimated to reach 85% [23,24]. During the 2022 MPXV outbreak, the rate of cases reporting smallpox vaccination ranged from 2% in a UK cohort of 156 cases [25], to 7% (3 out of 41 cases) in a Portuguese hospital cohort of patients with MPXV infection [26], and to 18% in a Spanish multicenter cohort (32 out of 181) [27]. 

In the present study, we evaluate the presence of residual cross-immunity against MPXV due to historic smallpox vaccination in a cohort of healthy donors. Understanding the level of immunological markers may inform public health policies and improve knowledge on antibody- and T-cell-driven protection against MPXV, an emerging virus with epidemic and pandemic potential.

## 2. Materials and Methods

One-hundred-eight healthy donors (HDs) were prospectively recruited for the study (70/2018; 43z del Registro delle Sperimentazioni Non Covid 2022 amended with approval 11z del Registro delle Sperimentazioni Non Covid 2023; approval 2z del Registro delle Sperimentazioni 2023). Seventy-one subjects were born before 1974 and reported smallpox vaccination during childhood; the median age was 58 years (IQR: 53–65; range 48–70; female/male: 44/27); 37 individuals were born after 1974 and were not vaccinated with VACV-based vaccines, and the median age was 32 years (IQR: 28–36.5; range 22–47; female/male: 23/14). One hundred and four individuals were health care workers, and four were blood donors from San Camillo Hospital in Rome. Informed consent for participation was collected from all the participants. Healthcare professionals included in the analysis were not exposed to Mpox-infected patients. The four blood donors reported no exposure to infected individuals.

Serum samples were available from 67 smallpox-vaccinated and 30 unvaccinated participants and were tested for the presence of anti-MPXV IgG and neutralizing antibodies. The median age was 59 years (IQR: 53–65; range 48–70; female/male: 42/25) for the smallpox-vaccinated and 31 years (IQR: 28–35; range 22–47; female/male: 19/11) for the unvaccinated. Peripheral blood mononuclear cells (PBMCs) were collected from 20 smallpox-vaccinated and 10 unvaccinated participants. The median age was 56 years (IQR: 53–60; range 49–67; female/male: 14/6) for the smallpox-vaccinated and 32 years (IQR: 29–44; range 25–47; female/male: 6/4) for the unvaccinated.

A viral stock was produced by infecting VeroE6 cells (ATCC, American Type Culture Collection, Manassas, VA, USA) with MPXV isolated in May 2022 from a skin lesion of a patient (GenBank: ON745215.1, referred to as the clinical sample). An indirect immunofluorescence assay (IFA) for anti-MPXV IgG was performed using home-made slides containing MPXV-infected VeroE6 cells, as described elsewhere [28]. To assess the presence of antibodies neutralizing the MPXV, serum samples were heat-inactivated at 56 °C for 30 min, and then, the plaque reduction neutralization test (PRNT) 50 assay was performed by incubating a 4-fold serial dilution (1:10 to 1:640) of serum samples with 100 infectious doses (TCID50) of the MPXV stock (2 h at 37 °C with 5% CO_2_) and putting the serum–virus mixtures in contact with 80%-confluent VeroE6 cells in 96-well plates. Each experiment contained replicates of cells with virus only (CV) and cells not infected in the presence of the specific serum. A viral titration was performed in parallel in each experiment as a control of viral input. Cells were observed each day to check the viral titer and cytopathic effect. On day 5, cells were stained and fixed with a solution of 90% crystal violet and 10% formaldehyde for 40 min. The cells were then washed twice with DPBS-1X and let dry. After counting the plaques with the Cytation 5 reader ( Biotek, Taranto, Italy), Nab titers were evaluated, and 50% protection was calculated using the CV as a reference.

The frequency of T-cell-specific responses to different OPXV and MPXV peptide pools was assessed via standard interferon-γ ELISpot. Briefly, PBMCs were isolated by using Ficoll density gradient centrifugation (Pancoll human, PAN Biotech, Aidenbach, Germany) and suspended in complete RPMI-1640 medium, with 10% fetal bovine serum, 1% L-glutamine, and 1% penicillin/streptomycin (Euroclone S.p.A, Pero, Italy). PBMCs were plated at 3 × 10^5^ cells per well in ELISpot plates (Human IFN-γ ELISpot plus kit; Mabtech, Nacka Strand, Sweden), stimulated for 18–20 h with pools of peptides from OPXV and MPXV (previously reported in Ref. [29]) and aCD28/aCD49d (1 µg/mL, BD Biosciences, Pero, Italy) at 37 °C (5% CO_2_). A T cell superantigen (SEB 200 nM, Sigma-Merck, Darmstadt, Germany) was added as a positive control. At the end of incubation, the ELISpot assay was developed according to the manufacturer’s instructions. Results are expressed as spot-forming cells per 10^6^ PBMCs (SFC/10^6^ PBMCs) in stimulating cultures after subtracting the background. The response to a peptide pool was defined as positive when the number of SFCs/10^6^ PBMCs measured was higher than the average + 2 standard deviations (mean + 2SD) of the SFC/10^6^ PBMCs observed in the unvaccinated individuals. 

## 3. Results

**Figure 1:**
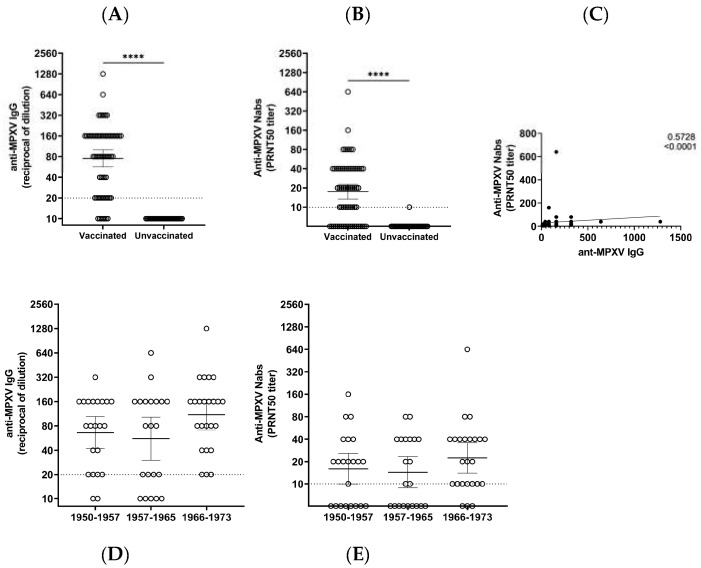
Levels of anti-MPXV IgG and Nabs in smallpox-vaccinated and unvaccinated HDs. (**A**) Anti-MPXV IgG and (**B**) Nab titers are expressed as the reciprocal of the serum dilution in 67 smallpox-vaccinated and 30 unvaccinated HDs. (**C**) Spearman r correlation analysis of anti-MPXV IgG titers obtained via IFA and Nab titers via PRNT50. Anti-MPXV IgG (**D**,**E**) Nab titers are expressed as the reciprocal of the serum dilution in smallpox-vaccinated HDs born in 1950–1957 (*n* = 22), 1958–1965 (*n* = 21), and 1966–1973 (*n* = 24). Dot lines represent the limit of the detection for IgG and Nabs (**A**,**B**,**D**,**E**). Statistical analysis was performed based on the Mann–Whitney test, **** *p* < 0.0001 (**A**,**B**,**D**,**E**), and Spearman test (**C**). For values below the limit of the detection, an arbitrary titer corresponding to half of the values of the detection limit was assigned. (**A**,**B**,**D**,**E**) Each circle represents a serum sample from an HD.

In the smallpox-vaccinated cohort, anti-MPXV IgG was detected in 60 individuals (89.6%), while 40 (70.1%) of them had Nabs (Figure 1A,B, Table 1). The geometric mean titer (GMT) was 75.2 (95% CI: 56.7–99.7) for IgG and 17.5 (95% CI: 13.4–22.8) for Nabs. Specifically, in the vaccinated cohort, we observed variable levels of IgG and Nabs, from near the detection limit to high titers reaching a 1:1280 serum dilution for IgG and 1:640 serum dilution for Nabs. All vaccinated individuals presenting Nabs also had anti-MPXV IgG. In the non-vaccinated cohort, anti-MPXV antibody levels were always below the detection limit, apart from one individual with weak reactivity in the neutralization test (1:10 serum dilution) who nevertheless did not present with MPXV IgG. When assessing the inter-assay correlation between IFA and PRNT50, we observed a highly significant correlation (Spearman r value: 0.57; *p* < 0.0001; Figure 1C). There was no significant correlation between age and anti-MPXV IgG (Spearman r value: −0.20; *p* = 0.098) or the Nab level (Spearman r value: −0.15; *p* = 0.218) in the vaccinees. To further investigate if the time from vaccination affected the humoral cross-immunity to MPXV, we analyzed the MPXV binding and Nab titers in the vaccinated HDs stratified in three groups as born between 1950 and 1957 (*n* = 22), between 1958 and 1965 (*n* = 21), or between 1966 and 1973 (*n* = 24) (Figure 1D,E). In these groups, serum samples were collected approximately >60 to 42 years after vaccination.

Even if higher MPXV antibody levels were observed in HDs born between 1966 and 1973, no statistically significant differences were observed when comparing the three groups for either anti-MPXV IgG or Nab titers (Figure 1D,E and Table 1).

Nevertheless, a higher frequency of individuals positive for IgG or Nabs was observed in the younger group of vaccinees when compared to the individuals born between 1958 and 1966 (Fisher exact test *p* = 0.017 and *p* = 0.040, respectively) or born between 1950 and 1965 (*p* = 0.044, *p* = 0.026) (Supplementary Table S1).

Subsequently, to evaluate the presence of an MPXV-specific T cell response, we stimulated PBMCs from 20 smallpox-vaccinated and 10 unvaccinated subjects with four different peptide pools, OPXV-CD4-E, OPXV-CD8-E, MPXV-CD4, and MPXV-CD8, and assessed the response by performing an IFN-γ Elispot assay. The peptide pools were previously designed based on experimentally defined Orthopox T cell epitopes or predicted T cell epitopes from the most immunodominant ortholog proteins of MPXV; the list of the peptides used in this study has been previously described [29]. It has already been demonstrated that the responses to these peptide pools are associated with VACV-specific T cell responses in a cohort of Dryvax-vaccinated individuals, Jynneos-vaccinated individuals, and MPOX convalescent individuals [29,30,31]. 

**Figure 2:**
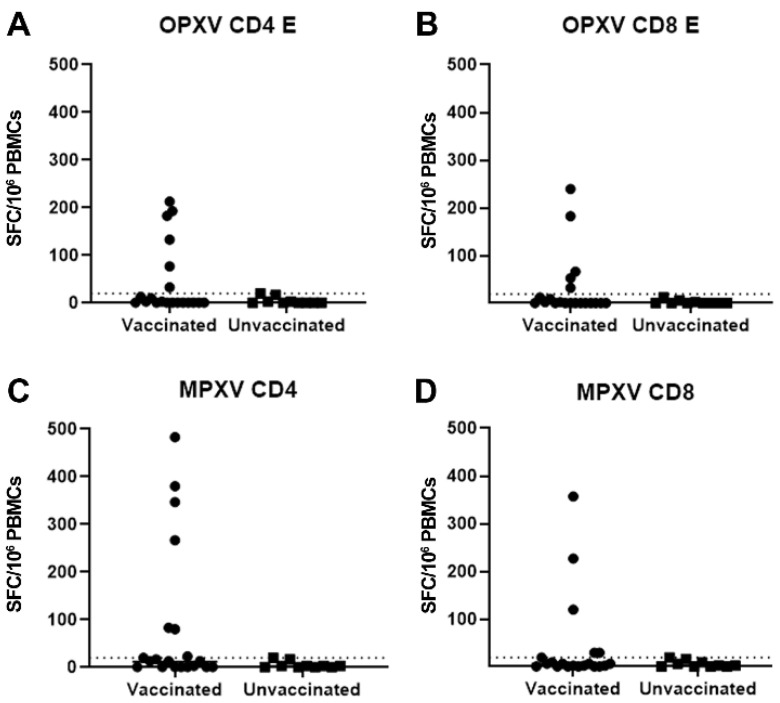
T cell response versus OPXV and MPXV peptide pools in smallpox-vaccinated and unvaccinated individuals. (**A**–**D**) T cells responses versus OPXV-CD4 (**A**), MPXV-CD4 (**B**), OPXV-CD8 (**C**), and MPXV-CD8 (**D**) peptide pools. IFN-γ-producing T cells are shown as spot-forming cells per million PBMCs (SFC/10^6^ PBMCs).

Our results showed a T-cell-specific response to OPXV-CD4-E and MPXV-CD4 in 6/20 (30%) of smallpox vaccinated HDs (Figure 2A,C). Five (25%) HDs also showed a response to OPXV-CD8-E and MPXV-CD8 pools (Figure 2B,D). 

A correlation between the magnitude of the T-cell-specific response to the OPXV-CD4-E and age was observed (Spearman r = −0.466, *p* = 0.038). When stratifying by date of birth, only one out of seven vaccinated individuals born before 1966 showed an OPXV- or MPXV-specific T cell response. All the other responders, 5/13 for OPXV-CD4-E or MPXV-CD4 and 4/13 for OPXV-CD8-E and MPXV-CD8, were born between 1966 and 1973.

## 4. Discussion

MPXV is a zoonotic virus of the Orthopoxvirus genus, which, on 23 July 2022, during the global outbreak, was declared a Public Health Emergency of International Concern. The study of the immunological markers of the response to OPXV in the human population has mainly focused on immunity to smallpox. Despite the increase in publications on MPXV infections and immunity observed in the last year [32], a limited number of reports focus on the response to the MPXV infection, and the level of cross-protection from MPXV due to vaccinia virus-based vaccines needs to be further investigated. We evaluated the presence of MPXV-binding and neutralizing antibodies and reactive T cells in a population of healthy donors with a history of smallpox vaccination. We observed anti-MPXV IgG and Nabs in a high proportion of tested individuals, 89.6% and 70.1%, respectively.

Despite the sample size presented here being not wide enough to estimate the prevalence of anti-MPXV humoral markers in the general population, the data are consistent with previous reports performed on even smaller cohorts [21,33,34]. Indeed, consistent with our data, a recent paper measured the levels of anti-MPXV Nabs in 19 individuals born before 1974, showing reactive Nabs in 79% of the serum tested [34]. Another study demonstrated a correlation of the number and area of plaques with the vaccination status by testing serum samples collected from 180 HDs (89 vaccinated, 96 unvaccinated); nevertheless, the neutralizing and binding activity was not titrated, nor was a clear cut-off shown, and no correlation between anti-MPXV A29L IgG and Nabs was shown [33]. Differently, a recent report described the absence of MPXV Nabs in 23 HDs who received smallpox vaccination 34–55 years ago [21]. In our cohort, we observed variable levels of Nabs, from near the detection limit to high titers (1:640 serum dilution). Whether the levels of Nabs measured can be considered protective upon infection or vaccination is unknown. Nevertheless, previous studies have shown that smallpox vaccination may confer protection against Mpox [23,24]. Of note, the mean titers of IgG and Nabs measured in our cohort of smallpox-vaccinated individuals are much lower when compared to the levels observed in a population of 18 acutely Mpox-infected individuals tested at our institute, where at 3 weeks post-infection, we observed a mean titer of 2826 for anti-MPXV IgG and 107 for specific Nabs [33]. With the exception of one case of weak reactivity in PRNT50 observed in one single unvaccinated participant, we did not observe the presence of MPXV-reactive antibodies in the unvaccinated population. The low reaction in the PRNT50 in the unvaccinated individual was not due to MPXV-reactive antibodies since the immunofluorescence test result was negative. The lack of anti-MPXV IgG in the unvaccinated population seems to exclude an unrecognized exposure to the MPXV or a closely related OPXV.

Regarding the persistence of the cellular immune response, a slow decline with time from vaccination and a half-life of 8–15 years of anti-viral T cells have been suggested in a previous study [17]. Compared to residual humoral cross-immune responses, we here report the detection of MPXV-cross-reactive T cells in a smaller proportion of historically vaccinated subjects, ranging from 25 to 30% of tested individuals. 

In previous publications, variable percentages (from 0 to about 80%) of responsive T cells upon stimulation with VACV or VACV-derived peptides have been described, and this variability may certainly depend on the laboratory protocol used and the assay selected [12,17,18,19,21,29,30]. The analysis of the T cell response was restricted to a subset of individuals because of the availability of whole blood samples for PBMC preparation from only 20 vaccinated and 10 unvaccinated participants. 

To avoid contrasting results, analyses based on a larger population and the use of standardized tests should be applied to better investigate the rate at which individuals show MPXV-cross-reactive T cells. The definition of a positive response applied here does not take into consideration the possible unknown exposure of unvaccinated individuals to OPXVs other than MPXV or VACV, and this could eventually underestimate the percentage of individuals with cross-reactive T cells. The unrecognized previous exposure to other OPXVs may also affect the humoral response.

The identification of participants in this study as smallpox-vaccine-vaccinated or unvaccinated is based on self-reporting and the year of birth, which may represent a limitation of the analysis, a limitation linked to the difficulties in tracing official documents of vaccination released over 40 years ago.

The availability of commercialized and standardized assays for the evaluation of MPXV humoral and cellular immunity markers would be of great help in defining the prevalence of specific antibodies and reactive T cells in different populations, i.e., historically and newly vaccinated subjects, acutely infected or convalescent patients, or immunocompromised individuals. 

The lack of a formally proven correlate of protection represents an obstacle to the interpretation of laboratory results and the definition of protective levels of virus-specific immune markers; nevertheless, the collection of laboratory data on immune markers is of paramount importance to achieve this goal. Ongoing clinical and epidemiological studies will be of help to define the level of protection induced by novel and historical vaccinations or infections [35,36,37].

Despite the limitations, we confirm the differential dynamics and persistence of humoral and cellular responses in historically smallpox-vaccinated people. Studies on a larger population will be necessary to deepen our knowledge on the duration of immunity and cross-immunity upon vaccination.

Data on population humoral and cellular immunity, which are derived from laboratory and epidemiological analyses of both infected and vaccinated individuals, may inform management plans for MPXV infections, outbreaks, and vaccination campaigns.

## Figures and Tables

**Table 1:** Anti-MPXV IgG and Nab titers and rate of positivity in smallpox vaccinated HDs.

	Born between (Years)		
	1950–1957	1958–1965	1966–1973
N.	22	21	24
IgG GMT (95%CI)	66.2 (41.9–104)	55.6 (30.2–102.6)	109.9 (71.4–169.2)
IgG % reactive (N)	90.1 (20)	76.2 (16)	100 (24)
Nabs GMT (95%CI)	16.0 (10.0–25.9)	14.4 (8.9–23.4)	22.5 (14.0–35.9)
NAbs % reactive (N)	63.6 (14)	57.1 (12)	87.5 (21)

GMT: geometric mean titer; Nabs: neutralizing antibodies.

## Data Availability

The raw data supporting the conclusions of this article will be made available by the authors, without undue reservation and upon request to the corresponding author.

## Supplementary Materials

The following supporting information can be downloaded at: https://www.mdpi.com/article/10.3390/vaccines11101541/s1, Table S1: anti-MPXV IgG and Nabs titre comparison after stratification based on HDs year of birth.
