# Supplementary material for: Evaluation of Cross-Immunity to the Mpox Virus Due to Historic Smallpox Vaccination
Source: Vaccines (Basel). 2023 Sep 28;11(10):1541. doi: 10.3390/vaccines11101541 (PMC10610801; doi:10.3390/vaccines11101541)
Supplement: Supplementary file 1 [file vaccines-11-01541-s001.zip › vaccines-2591955-supplementary.pdf]

Supplementary Table S1 anti-MPXV IgG and Nabs titre comparison after stratification based on HDs year of birth

Born between (years)	IgG reactive (N)	Ig G Fisher test vs 1950-1957	IgG Fisher test vs 1958-1965	IgG Fisher test vs 1950-1965
**1950-1957**	20 (22)	n.a	p=0.240	n.a.
**1958-1965**	16 (21)	p=0.240	n.a	n.a.
**1966-1973**	24 (24)	p=0.220	p=0.017	p=0.044
**Born between (years)**	**Nabs reactive (N)**	**Nabs Fisher test vs 1950-1957**	**Nabs Fisher test vs 1958-1965**	**Nabs Fisher test vs 1950-1965**
**1950-1957**	14 (22)	n.a	p=0.759	n.a.
**1958-1965**	12 (21)	p=0.759	n.a	n.a.
**1966-1973**	21 (24)	p=0.081	p=0.041	p=0.026

Abbreviations. HDs: healthy donors; Nabs: Neutralizing Antibodies; n.a.: not applicable
